# Supplementary material for: Carbon Nano-Fiber/PDMS Composite Used as Corrosion-Resistant Coating for Copper Anodes in Microbial Fuel Cells
Source: Nanomaterials (Basel). 2021 Nov 21;11(11):3144. doi: 10.3390/nano11113144 (PMC8622003; doi:10.3390/nano11113144)
Supplement: Supplementary file 1 [file nanomaterials-11-03144-s001.zip › nanomaterials-1448847-supplementary.pdf]

Supporting Information

Carbon Nano-Fiber/PDMS Composite Used as Corrosion-Resistant Coating for Copper Anodes in Microbial Fuel Cells

Fatma Bensalah [1,2], Julien Pézard [1], Naoufel Haddour [1,*], Mohsen Erouel [2], François Buret [1] and Kamel Khirouni [2]

[1] Laboratoire Ampère, Ecole Centrale de Lyon, 36 Avenue Guy de Collongue, 69134 Ecully, France; naoufel.haddour@ec-lyon.fr
[2] Laboratory of Physics of Materials and Nanomaterials Applied at Environment, Faculty of Sciences in Gabes, Gabes University, 6072, Gabes, Tunisia; khirouni.kamel@gmail.com
* Correspondence: naoufel.haddour@ec-lyon.fr

Keywords: microbial fuel cell; anode materials; copper electrodes; CNF-PDMS; surface modification; biofilm

1- MFC setup and sampling

MFC bottles MFCs with a "classical" configuration were used (Figure S1). The cathode was an air cathode created by following the procedure of Cheng et al. [124]. It contains one catalytic slide (the inner side) and one diffusion side (the outer side). The catalytic side is composed of carbon powder with 5% platinum powder. The diffusion side is composed of 4 layers of PTFE (Polytetrafluoroethylene). The anode consisted of bare Cu or CNF-PDMS Cu coated anodes

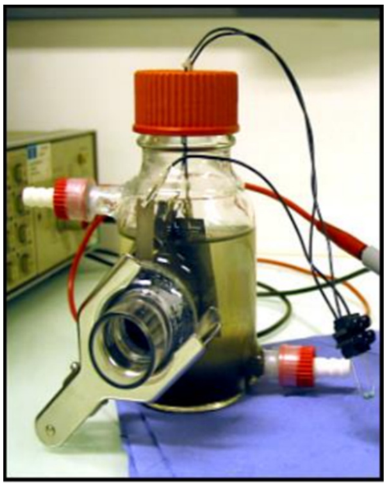

Figure S1. MFC bottle with an air cathode.

2- Interfacial capacitance

The interfacial capacitance was estimated from the ratio between capacitive current and the scan rate (see SI). The capacitive current is determined from the current difference between the forward and backward sweeps. This current is measured for different scan speeds. By plotting capacitive current as a function of the scanning speed, a straight line is obtained, the slope of which corresponds to the value of the interfacial capacitance C_{dc} of the electrode. In fact, the capacitive current I_{dc} is directly proportional to the scanning speed ν according to Equation :

$$I_{dc} = C_{dc}.\nu$$

3- Electrical performance of MFCs

Electrode	Open Circuit Potential (mV)	Maximum Power Density ($mW.m^{-2}$)	Maximum Current Density ($mA.m^{-2}$)
Cu	163 ± 11	8 ± 1	179 ± 9
Cu-CNF-PDMS (250 μm)	444 ± 38	35 ± 4	332 ± 24
Cu-CNF-PDMS (500 μm)	450 ± 6	70 ± 8	559 ± 80
Cu-CNF-PDMS (1000 μm)	457 ± 5	50 ± 3	403 ± 40
